# Characterization of lead sulfide obtained from *Naga Bhasma*

**DOI:** 10.1016/j.jaim.2023.100864

**Published:** 2024-03-24

**Authors:** Manoj Kumar Dash, Namrata Joshi, Laxmikant Dwivedi, Vd Sushil Dubey, Kamal Nayan Dwivedi

**Affiliations:** a Dept. of Rasashastra, Govt. Ayurveda College, Raipur, C.G, India; b Dept. of Rasashastra, Faculty of Ayurveda, IMS, Banaras Hindu University, Varanasi, 221005, India; c National Institute of Ayurveda, Jaipur, Rajasthan, India; d Dept. of Kriya Sarira, Faculty of Ayurveda, IMS, BHU, Varanasi, India; e Dept. of Dravyaguna, Faculty of Ayurveda, IMS, BHU, Varanasi, India

**Keywords:** *Naga bhasma*, Characterization, XRD, SEM, TGA, DTA, FTIR

## Abstract

**Background:**

Lead sulfide nanoparticles were manufactured from lead oxide using a procedure described in the Ayurveda formulary of India, which involved using a quantum of the heat of up to 60 puta, which is officially known as the *Shasti puta Naga Bhasma*.

**Objective:**

The study shows sulfurization of nanoparticles significantly decreased their toxicity due to the lower solubility.

**Materials and Methods:**

The present work used the arsenic sulfide media and traditional puta for processing and the characterization of the same has been conducted. Different analytical techniques like X-ray diffraction (XRD), scanning electron microscopy (SEM), energy-dispersive X-Ray (EDX) spectroscopy, Fourier transform infrared spectroscopy (FTIR), and thermo-gravimetry analysis (TGA) were used.The study shows sulfurization of nanoparticles significantly decreased their toxicity due to the lower solubility. Materials and Methods: The present work used the arsenic sulfide media and traditional puta for Naga bhasma processing and the characterization of the same has been conducted. Different analytical techniques like X-ray diffraction (XRD), scanning electron microscopy (SEM), energy-dispersive X-Ray (EDX) spectroscopy, Fourier transform infrared spectroscopy (FTIR), and thermo-gravimetry analysis (TGA) were used.

**Results:**

Powder x-ray diffraction, scanning electron microscopy, Fourier transform infrared spectroscopy, Thermogravimetric analysis, and differential thermal analysis all showed that the produced nanoparticles are lead sulfide nanoparticles with a particle size of an average of 84.60 and the crystalline average size of 69.06 nm.

**Conclusion:**

The rounded, rod, oval, cubic, and circular morphology of the produced lead sulfide nanoparticles can be seen in the SEM image. The stretching and bending functional groups in the sample were alkanes, alkenes, aromatic hydrocarbons, aromatic hydrocarbons, carboxylic acids, alkyl carboxylic acids, alkyl alpha, beta-unsaturated, aldehydes, ketones, carboxylic acid, aliphatic amines, primary amines, secondary amines, alkyl halides, alkyl halides are studied through the FTIR spectrum.

## Graphical abstract

**Figure:**
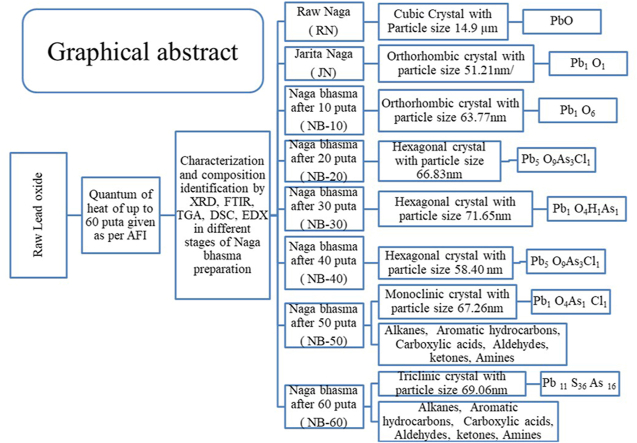

## 1 Introduction

The calcined ashes, technically designated as *Bhasma*, are the particular dosage form pertinent to the Traditional system of medicines in India. The repeated incineration with a particular quantum of heat before which impregnation with herbal juices is carried out remains behind each calcination while the processing of *bhasma* is carried out. Here, the calcined lead, traditionally labeled as *Naga Bhasma* (NB) was prepared which has targeted *Rasayana* properties [1] and used in combination with different herbal formulas or even as single doses. Classical quotes contemporize its uses in diseases like diabetes mellitus, various urinary disorders, rheumatoid arthritis, etc [2]. Recent research substantiates the properties of this particular medicine in the regeneration of germinal epithelium of testes, evidence of cure for diabetes mellitus, etc [3]. The heavy metal particularity and the conventional hesitations behold the prescription of the NB, even though practiced and propagated since ancient times. Pharmaceutical processing greatly influences the outcome of this type of incinerated preparation. The manufacture of *bhasma* starts from peculiar procedures called *Shodhana,* the purificatory methods, *Bhavana,* the impregnation process, *Marana,* the calcination episodes, and finally a set of examinations called *bhasma pareeksha* which would finalize the dosage form applicability to biological systems. Table 1 provides a detailed overview of the already published green protocols for the synthesis of lead sulfide. Based on the properties, characterization lead sulphide prepared from Green protocols viz; *Aloe vera* extract (Potential hole transport layer for high-efficiency perovskite solar cell applications) [4]; Lead exposed to yeast *Rhodosporidium diobovatum* confirmed the presence of PbS nanoparticles of cubic structure [5]; Red green blue (RGB) photoluminescences (Impressive open circuit voltage) [6]; Aqueous extracts of roasted coffee, fruit juices or carbon-containing natural products (used for diagnosis of SARS-CoV-2 for pandemic control) [7]; l-cysteine-desulfurizing bacterium Lysinibacillus sphaericus SH72 (Coats the surface of PbS nanoparticle as a stabilizing ligand) [8]; Moderate halophile, *Idiomarina* sp. strain PR58-8 (targeted bioimaging via modifying the surface of the particles) [9]; Glutathione and 3-MPA (mercaptopropionic acid) as the stabilizing ligand using a green approach(Excellent fluorescence stability and very low cytotoxicity in normal kidney cell and cancerous HeLa cell.) [10]; Gum Arabic PbS NPs to the hypersaline unicellular green algae *Dunaliella salina* (reduces the toxicity of lead nanoparticles) [11]. The above methodology shows they are suitable for catalysis, imaging, medical applications, and environmental applications whereas Lead sulfide prepared from NB is reported to have biological activities. Importantly,NB-prepared Lead sulfide has essential functional groups, safe in histopathology studies on rats, anti-diabetic effect.

Given that they typically have lower bioaccessibility and absorption rates than ambient pollutants, it is not unexpected that metals in their sulfide forms have reduced potential toxicity. Recent studies in this area have a lot of interest in the oxidation of sulfurides. The freshly formed surface oxidation layer alters the chemical structure of the virgin material and is prone to further rearrangement under reaction conditions. Modern organic and organometallic chemistry relies heavily on transition metal catalysts because of their intrinsic properties, such as changing oxidation state (oxidation number), generation of complex ions, and catalytic activity. The whole processing necessitates the submission of manpower, money, resources, etc. and so the entire operation behind the outcome can be categorized as a tedious one. The recent developments signify this lengthy course of action results in particle size reduction, conversion of oxidation state, the addition of certain functional groups, the addition of trace elements, and so on. NB (nanoparticle) morphology (size, shape, and composition) and surface chemistry are the determining factors underpinning the efficacy of such materials in therapeutic applications. The size, shape, and surface chemistry of naga bhasma (nanoparticle) can strongly influence key properties such as interactions with diverse biological fluids and interfaces and, in turn, impact the delivery of bioactive load, modulating therapeutic performance. NB in addition to treating diabetes mellitus, has been prescribed for certain disorders related to the liver, spleen, and skin. SEM analysis Table 2, Fig. 3a, Fig. 3b, Appendix A) provides a detailed size distribution of NB. with increasing puta, there is a change in the shape from less to more from spherical - to– cubic-to- oval -to rod and varies in size from 14.9 μm to 69.06 nm. NB-60 provides a synergistic effect that enhances the positive treatment outcome. Change in shape from less to more from spherical to rod with a size of about 69.06 nm exhibits improved cellular uptake, intracellular processing, and transport through tissues and organs [12]. Rod shapes were known to be taken up by macrophages at faster rates than spherical particles [13]. Spherical nanoparticles have a higher level of α-amylase and α-glucosidase inhibitory activities [14]. Also, it promotes the wound-healing process effectively [15].

The NB was also subjected to these procedures which made the end product biocompatible with the deletion of untoward effects of the metal part. NB is analyzed here in detail during different stages of pharmaceutical processing both conventionally and according to the classical versus with hypothesizing on the conversion of toxic lead to medicinally effective NB with the above described pharmaceutical policies undergone by it. So this paper can be taken as a fingerprint of differences attaining behind each pharmaceutical processing in the conversion of metal lead to *Naga Bhasma,* the quality assessment, reduction of intermediate process substantiating towards discrepancies, etc.

NB can be manufactured in 31 different methods including the mercury-sulfur (*Parada-gandhaka*) media method (mostly in 7 *putas*) [4], realgar (*manashila*) media method (mostly 60 *putas*) [4], poisonous herbs media method (mostly 6 *putas*) [4], only plant media approach (100 *putas*) [4] and alkali (*kshara*), salt *(lavana*) media methods (*Gaja puta*) [16]. The arsenic disulfide combined preparation process stands out commonly among the 31 different references and the quantum of heat varies from the 3 *puta* to 60 *puta.* The very present method has been practically validated as feasible in the attainment of the qualified *bhasma* with *niruttha* property (inability to regain metallic form). The present work also has used the arsenic sulfide media and traditional puta forNB processing and the characterization of the same has been done through different analytical techniques like X-ray diffraction (XRD), scanning electron microscopy (SEM), energy-dispersive X-Ray (EDX) spectroscopy, Fourier transform infrared spectroscopy (FTIR), and thermo-gravimetry analysis (TGA)

The coding of different tested samples is as follows.

RN-Raw *naga*, SN-*Shudha naga*, JN*-Jarita naga*, NB10-*Naga bhasma* 10 *puta*, NB20- *Naga bhasma* 20 *puta*, NB30- *Naga bhasma* 30 *puta*, NB40- *Naga bhasma* 40 *puta*, NB50- *Naga bhasma* 50 *puta*, NB60-*Naga bhasma* 60 *puta*, MS60-*Manashila satva*.

## 2 Material and methods

The raw ingredients lead and arsenic disulfide (*manahshila)* were of analytical standards procured from the Jaipur market, Rajasthan.and were checked for the lead(*naga*) and the arsenic portion respectively. Cow's urine was from the *goshala,* Jaipur, and the fruits of *Tamarindus indica* L. (*Chincha*)*,* the bark of *Ficus religiosa* L. (*ashwatha),* seeds of *Dolichos biflorus, Zingiber officinale* Roscoe, buttermilk was purchased from the local market.

### 2.1 Pharmaceutical processing of NB

The processing was followed as described in the Ayurveda formulary of India with a quantum of heat up to 60 puta officially marked as the *shasti puta* NB methodology [17].

#### 2.1.1 Purification (*shodhana*) of As_2_S_2_ (*manahshila*)

The preparatory stages were initiated with the *shodhana* process/purificatory processing which in the case of the realgar/arsenic disulfide/*manashila* was the trituration with the liquid extract of *Zingiber officinale* Roscoe till the mixture was dried and the repetition of the same seven times. The 4 Kg realgar was subjected to the above-mentioned processing using up to 3600 ml ginger extract as liquid and the final material weighed up to 4340 gm with a percentage gain accounting for 108 % [18].

#### 2.1.2 Purification method (*Samanya shodhana*) of *naga*

Purification to *Naga* was done with melting initiated in a long-handled ladle with 500 gm of the material and the immediate quenching in five different liquid media viz. oil of *Sesamum indicum* L.; buttermilk; sour gruel; cows urine; decoction of *Dolichos biflorus* L., three times. The final product was a granular greyish color resultant with 440 gm in weight expressing a yield of 88 % [19].

#### 2.1.3 Jarana

The processed *Naga* was again melted in a flat iron pan and fine powders (80 mesh) of *Ashwattha* (*Ficus religiosa* L.) and *Chincha* (*Tamarindus indica* L.) were sprinkled over the molten lead (approximately 10g–15g in each attempt). Each time addition of powders has to be followed with continuous mixing up of molten metal preferably with an iron ladle. The pressure imparted and friction generated with the mixing-up process helps in the merging of the herbal and the metal powders. The herbal portion eventually burns up completely and each fresh addition was only after completing the burning of prior added powders. The temperature was controlled up to 350–400 °C till all the material turns up to ash and was later covered with an earthen plate. The temperature was then hiked up to 650–680 °C and maintained the same for 4 h. The whole resultant, technically the *Jeerna Naga* (JN), on cooling was washed with a solution of hot water and sour gruel (*Kanji/*fermented acidic preparation) to separate the alkaline proportion. The washing was done 3 times till neutral pH was attained and the whole end product turned out to be 490 gms with a %gain of 111 % [20].

#### 2.1.4 Marana

The arsenic disulfide in an equal proportion of 250 gm was mixed with the obtained JN and levigated with sour gruel for 3 h. The processed mass of material was made to flat, round pellets each 15–30 mm in size, and kept for drying ((Fig. 1a).

**Fig. 1a:**
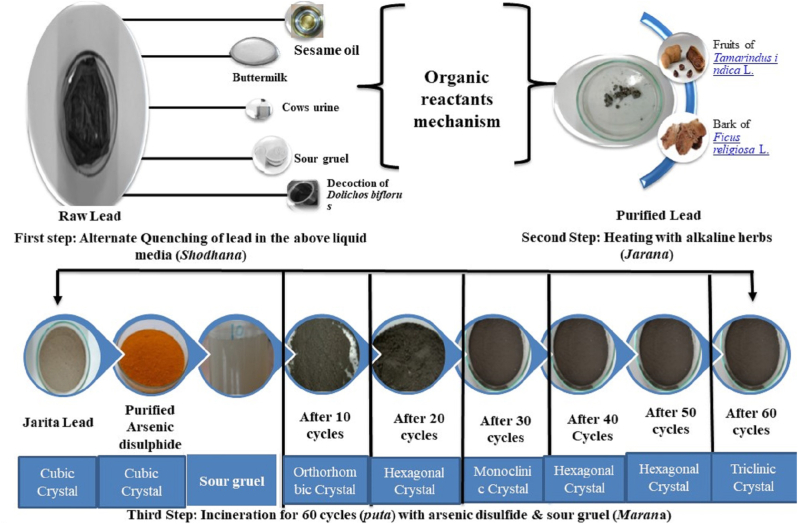
Organic reactants mechanism and the present nanostructure in the synthesis of NB

The dried ones were transferred to an earthen saucer and covered with an earthen saucer with approximated edges and the same size. The joining ends were sealed with clay and a well-dried pair of earthen saucers was kept for calcination purposes [21]. The classical version of fulfillment criteria which satisfy the *varna* (color), *varitaratva* (floats on water), *rekhapurnatva* (which enters ridges of the fingers when rubbed between), *sukshmatva* (extreme fineness), and *nishchandratvam* (loss of metallic luster), *apunarbhava* (inability to regain metallic form), *niruttha* (inability to regain metallic form) was studied (Fig.1b). The precisely examined above points were included at different stages of the manufacturing process. The *Apunarbhava* examination includes the processing of the NB 60 with a group of materials called *Mitrapanchak* [22]*.* An equal proportion of jaggery, *Abrus precatorius* L., borax, honey, and cow ghee is made to triturate with the calcined end product, the NB 60. The mashed-up mass is later subjected to drying up after making round pellets as before. The temperature of 700 °C for 3 h was made to observe the changes and check for metallic from regaining. There were no such changes in metallic characters. Redox of lead sulfides to elemental lead does not occur. The confirmatory process for *Apunarbhava's* examination with NB 60 was done by subjecting them to heat treatment with pure silver. The process is called as *Niruttah* examination and signifies the weight attainment in the silver metal as the active metallic part of the calcined product remaining as such. Redox of lead sulfide to elemental lead was not seen. The confirmatory process for *Apunarbhava's* examination with NB 60 was done by subjecting them to heat treatment with pure silver. The process is called as *Niruttah* examination and signifies the weight attainment in the silver metal as the active metallic part of the calcined product remaining as such. Here, the NB 60 didn't cause any change in weight for the pure silver. The measure of heat treatment is shown in Table 1. These whole procedures were repeated 60 times with 1/4th arsenic disulfide addition from the 2nd to 60th puta (NB 60) and examined for *Bhasma pareeksha* attainment. The heat treatment pattern was *ardhagajaputa in* the beginning till the 50th *puta* and *gajaputa* thereafter till the end and detailed in Table 1. In a total of 4.08 kg of arsenic disulfide, 6.9 L of sour gruel were used up and 290 gm of NB was obtained with a percentage yield of 116 %. The upper earthen saucer had a condensed underside collection of arsenic disulfide extraction (*manashila satva)* amounting to 112 gm.

**Fig. 1b:**
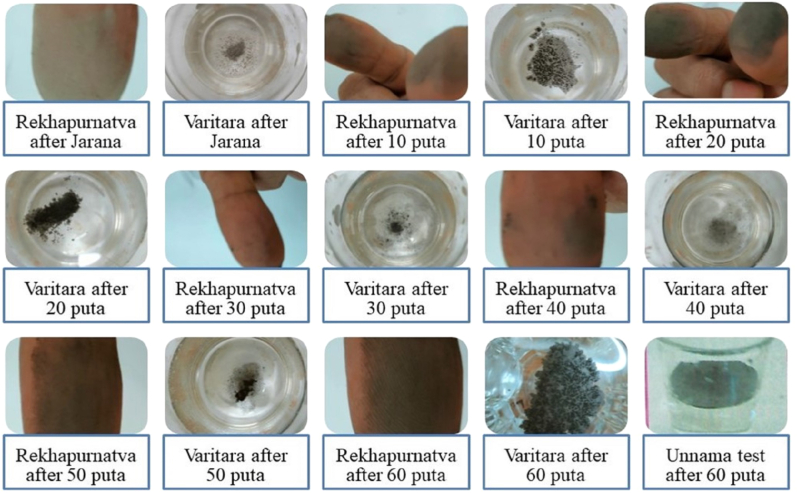
Fourth step: Ayurvedic attributes Chemical test

**Table 1:** Published Green protocols for the synthesis of lead sulphide

Sr. No	Method of synthesis	Green protocols	Characterization	Properties
1.	Chemical precipitation method	Aloe Vera extract with PbCl_2_ and Thiourea (H_2_N-CS-NH_2_)	XRD and TEM results confirm that the films are in the cubic phase	Potential hole transport layer for high-efficiency perovskite solar cell applications.
2.	Culture method	Yeast Rhodosporidium diobovatum	XRD confirmed the presence of PbS nanoparticles of cubic structure. Crystallite size as determined from transmission electron microscopy was found to be in the range of 2–5 nm	The lead-exposed yeast displayed a marked increase (280 % over the control) in nonprotein thiols in the stationary phase)
3.	Heterogeneous reaction Method	Red green blue (RGB) photoluminescences	High degree of crystallinity even for the extremely small sizes (1–3 nm)	Impressive open circuit voltage (∼0.8 V) beyond that reported to date.
4.	Green Natural products	Aqueous extracts of roasted coffee, fruit juices or carbon-containing natural products	NIR fluorescence 2–4 mm at 900–1900 nm	Used for Diagnostics of SARS-CoV-2 for pandemic control)
5.	Green bacterial biosynthesis	l-cysteine-desulfurizing bacterium Lysinibacillus sphaericus SH72	Cubic structure, and are often aggregated as spheroids of about 105 nm in size	Coats the surface of PbS nanoparticle as a stabilizing ligand)
6.	Chemical bath and Photochemical deposition methods	Moderate halophile, *Idiomarina* sp. strain PR58-8	Spherical β-PbS_2_ nanoparticles (PbS_2_NPs) with a tetragonal crystal lattice, a crystallite domain size of 2.38 nm, and an interplanar distance of 0.318 nm.	Targeted bio imaging via modifying the surface of the particles
7.	Green approach	Glutathione and 3-MPA (mercaptopropionic acid) as the stabilizing ligand using a green approach	Formation of profoundly immaculate and crystalline QDs	Excellent fluorescence stability and very low cytotoxicity in normal kidney cell and cancerous HeLa cell.
8.	Green method	Gum Arabic PbS NPs to the hypersaline unicellular green algae Dunaliella salina	Negative effect on aquatic algae and their transformation by GA capping affects NPs aggregation properties and toxicity.	Reduces the toxicity of Lead Nano particles

## 3 Characterization

The characterization of RN, SN, JN, NB 10, NB 20, NB 30, NB 40, NB 50, NB60, and MS 60 done here includes analytical examinations like powder X-ray diffraction (XRD), gravimetric analysis (TGA). A benchtop powder x-ray diffraction (XRD) diffractometer (Miniflex, Rigaku, Japan) operating at 10 kV and 15 mA was used for XRD analysis. EVO 18 Carl- Zeiss, Germany in the SEM analysis revealed the morphological particularities of the formed *bhasma.* FTIR spectra of the liquid precursor were recorded on a Thermo Nicolet model IS5 instrument (Thermo Fisher Scientific, USA), employing liquid precursor/gel/solid coatings on KBr pellets and not less than 32 scans were conducted for each resolution with 4 cm^−1^ was used for this. A thermogravimetric analyzer (TGA-50 Shimadzu Scientific Instruments, Japan) in a Nitrogen atmosphere revealed the changes corresponding to the temperature pattern adopted for the formation of the calcined end product.

## 4 Results and discussion

### 4.1 XRD phase analysis

**Fig. 2:**
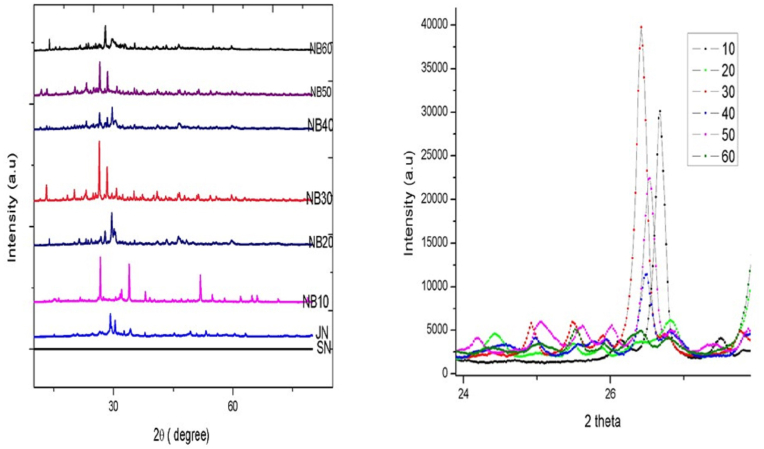
Powder X- Ray diffraction Patterns of NB in different Putas

The sample markings RN, SN, JN, NB-10, NB-20, NB-30, NB-40, NB-50 & and NB-60 represented XRD patterns are incorporated in Fig. 2. The above all exhibited highly crystalline structure which matches with the reported patterns as mentioned in JCPDS card numbers (JN: Pb1 O1, 003–6250); (NB-10: Pb_1_O_6_, 002–1074); (NB – 20: Pb_5_ O_9_ Cl_1_ AS_3_, 003–1863); (NB-30: PbAs1O4, 002–9552); (NB- 40: Pb As3 Cl 1O 9, 0031863); (NB-50: Pb_5_0_9_As3 Cl1, 003–1863); (NB- 60: Pb11 S36 As16, 001–8123); (M.S-60 As_4_O_6,_ 003–6144). JN crystals system shows Orthorhombic symmetry of space group Pbma have lattice constants of a = 5.48, b = 4.75, and c = 5.89, Volume of cell 153.76, number of molecules (Or formula units) present in the unit cell (Z) was 4 and Reference intensity ratio (RIR) was 6.60. NB-60 crystal system shows triclinic of space group p-1 have lattice constants of a = 22.7, b = 8.3, and c = 7.9, Volume of cell 1486.6, number of molecules present in the unit cell (Z) was 2 and reference intensity ratio (RIR) was 1.19. The crystal structural variations of various samples of NB are shown in Table 2. Every sample shows regular Pb metal peaks. The highest intensities were shown by the peaks (125) and (120) found in NB20, NB30, NB40, NB50, and NB60. The next highest intensity peaks were (112, and 111), which were seen in JN & NB10. The interplanar spacing increased from JN to NB60, according to a comparison of the results from the RN, SN, JN, NB10, NB20, NB30, NB40, NB50, & and NB60. RN to SN intensity ratios of various peaks showed a slight variation when incineration with arsenic disulfide at 700 °C was used, the intensity of the first peak, 004, increased significantly from 26.4 % to 100 %. The development of additional peaks in addition to the Pb peaks shows that the heating with arsenic disulfide significantly altered the crystal dimensions in some regions of the *Naga bhasma*. With arsenic disulfide, the intensities of these peaks increased, indicating a strengthening of crystal aberrations. From RN to NB60, the d spacing was somewhat reduced, which showed a reduction in the distance between atomic planes. In NB50 and NB60, broader peaks were seen, which is an indication of decreasing crystallite size since as crystallite size decreases, peak width increases. At large angles 2Theta, the crystallite size broadening is the most obvious. The quick fall in FWHM shown in NB60 is an indication of variations in microstructure and a reduction in tensile stress [23].

The cubic pattern for RN after the *jarana* process obtained the orthorhombic structure as per the analysis outcome. The orthorhombic pattern was continued up to 10 *puta* and thereafter the pattern changed to hexagonal for each analysis corresponding to NB-20, NB-30, and NB-40. The NB50 exhibited a monoclinic pattern and further heat treatment till NB60 revealed a triclinic crystal structure. Each calcination attempt 60 times gradually made the impure lead (PbO) be changed to Lead sulfide (Pb 11 S36) phase change with an additional Arsenic peak. The reference peak for Pb 11 S36 exhibits a triclinic crystal system with the presence of tetra arsenic oxide phases. The XRD phase analysis clarifies the difference in heat treatment in each calcination that brings forth the changes in the internal structure of the end product. The changes are overall inclusion from the processing, composition, amount, and distribution of phases, and grain boundaries finally reflecting upon the properties of each NB sample.

The initial phase of pharmaceutical processing, the *Samanya Shodhana,* with repeated cycles of heating and dipping in several media, made the conversion of the solid lead to coarse powder form. The whole processing which includes the heating till the critical melting point and the maintenance of the temperature followed by instant cooling in different media made the metal brittle. The cyclic discourse of the procedure made the brittleness to be enhanced till the end making the powder appearance. Here, the metal is subjected to heating and quenching in the open air and so the resultant action can be from the abundance of oxygen together with changes in the hardness and tensile strength of the carbon content after quenching [24]. The core metal gets conjoined with the oxygen during this repetition leading to the final compound form.

The contaminants present could have either oxidized or submerged in the liquid portion and the continuous replacement of liquid media after each quenching together with straining removes the unwanted portion with each successive step. The quenching also evidences the displacements of ionic crystals in the interstitial voids and the softening of the metal particulates by the oil/fat content of the respective media [25]. Among all 5 liquids, the cow's urine only exhibits the alkaline pH and the rest of the media are within the acidic range. This peak tilt of hydrogen ionic concentration and the differences in alternating media also contributed to the dissociation of the contaminants and the fragmentation of the core metal. The universal principle of alkalinity creates a disintegrating approach on metals and acidity creates the dissolution of impurities can be seen adopted in this *Samanya shodhana* also [26].

The procedure to *Jarana* can be regarded as a preparatory stage exclusively meant for a certain group of metals called *Putiloha* according to the pioneers in traditional medicine. The process is meant for the increment in the melting point of this category of metals as the group consists of metals with a low melting point. This type of heat treatment makes the metals prepared for the next step of calcination/*Marana* where intense and peak temperature patterns are implied. Conjoined heat treatment of these types of metals and herbals like *Ficus religiosa* L. & and *Tamarindus indica* L. make vacancies in pairs of anions and cations due to their alkaline nature [27]. In the course of the procedure, gradual cation voids are created in the lead metal with electron ejection. The oxygen charges on the metal directly to the lead oxide (Pb_1_ O_1_) formation and the slow conversion of the metal to fine powder form.

The final stage of pharmaceutical processing designated as *Marana* was carried out with As_2_S_2_ and sour gruel which all molded out the final compound concerning shape and microstructure. The pattern of heat treatment for the initial 50 *puta* with *ardhagajaputa* and later 10 *puta* with *gajaputa* made the triclinic crystals lead sulfide. *Ardhagajaputa* temperature pattern exhibited the following variations including 700 °C for 30 ± 5 min after ignition of *puta*, above 400 °C temperature was maintained for 30 ± 5 min, and above 200 °C temperature was maintained for 30 ± 5 min, and also self-cooling was found at 6 h duration.

The second phase of pharmaceutical processing (*Jarana)* which maintained the temperature range in a high proportion up to 630–680 °C for 4 h induced orthorhombic distortion to exist cubic symmetry. The third phase in comparison to this second phase didn't exhibit the peak temperature pattern duration in both heating styles-*ardhagajaputa* and *gajaputa.*

The gradual increase of temperature and consequent self-cooling (*Swangashita)* is applied in this concept of temperature application. The phase transition from orthorhombic crystals of *jarana* to hexagonal crystals of NB20 to NB40 can be considered as the application of the gradual decrease of the temperature. Since the uniform heating pattern and self-cooling method were adopted from NB-40 to NB-50, the phase transition to hexagonal monoclinic might be also due to the same temperature maintenance. The phase transition is initiated by increasing monoclinic short-range order (SRO) of the only partly ordered Pb ions within the hexagonal domains causing the rearrangement of the Pb and O ions towards the Hexagonal Symmetry in NB-40. The entire data can be summarized as an order-disorder phase transition from cubic-to-orthorhombic, then to monoclinic, and ending up in triclinic.

The *Gajaputa* maintenance of heating pattern includes 900 °C for 70 min after ignition of *Puta*, above 700 °C temperature was maintained for 55 ± 5 min, above 500 °C temperature was maintained for 80 ± 5 min, and also self-cooling time duration was found at 8 h 10 min. The shape determination of each sample depends on many factors such as internal symmetry, randomly oriented grains, and crystal defects. Atoms around the atomic site of different puta NB (Pb1 to Pb 11) are displaced, foreign atoms replacing and sitting in the parent atom. The grain boundary is responsible for grain growth on heating (Pb1 O1 to Pb 11 S36). On heating of As_2_S_2_ with lead, the grain boundary (Increase in the concentration of Pb at grain), and crystal orientation (Increase in calculated density) change abruptly. The change of the Low angle boundary of JN to the high angle boundary of NB-60 can be described as an array of dislocation. On heat treatment and self-cooling, RN changes its cubic crystal to triclinic texture in NB-60. This change can reintroduce therapeutic properties in *Naga bhasma*. In NB-60, Arsenic semimetals have charge carriers to overlap with the valence band. On heating an earthen vessel containing Arsenic disulfide with lead, arsenic sublimes and condenses as a shiny film a cooler part of the earthen vessel. Strong heating will decompose it into arsenic & and hydrogen. The shiny material was tested for XRD, SEM, TGA, and FTIR. The oxidation state of As is changed from its original +2 valence state to +4 valence state forming As_4_O_6_ having potential effects in cancer therapeutics. Sulfur plays an essential role in removing arsenic from *Naga bhasma*. The average crystallite size for Pb1 O1 and Pb 11 S36 was found to be around 50–70 nm as calculated by Scherer's formula in Table 2. During each puta, some material was sublimed and condensed Naga as a shiny film at the cooler part of the earthen vessel was collected (MS-60)

The reason behind this is the synthesis or degradation of triclinic crystalline structures in low-temperature, within the nano range, having the least toxicity in therapeutic dose.

After the *niruthha* and *apunarbhava* tests, no weight gain was observed. It is indicative that the proper formation of the Pb_11_S_36_ does not transform back to Pb and the sample regains its original straight shape. This is possible because NB-60 crystals originally formed a single crystal. But in NB-50 on heating with *Mitrapanchaka* or Silver the lead sulfide re-transforms (20 %), but its shape seen under SEM does not change due to the self-accommodated structures. The reason for such complex behavior is that the NB does not change its state when it is converted to triclinic sulfide form. The triclinic unit cell in NB-60 has the least-symmetrical shape of all unit cells while the monoclinic unit cell in NB-50 is distinguished by a single axis, called an axis of two-fold symmetry, about which the cell can be rotated by 180° without changing its appearance in high temperature [28].

### 4.2 Energy-dispersive X-ray spectroscopy (EDX)

The Energy-dispersive X-ray spectroscopy (EDX) employed analytical data to produce the Pb and O peaks in RN, whereas with NB60 the peaks were Pb, O, S, As, Na, Mg, Ca, C, and Fe as shown in Table 4. The additional peaks in the second sample suggest the contributions from *Ficus religiosa* L. and *Tamarindus indica* L. during *jarana* and As_2_S_2,_ sour gruel during *Marana*. The biocompatibility of bhasma dosage forms might be attributed not only to the core metal structure but also to additional elements introduced during the processing of bhasma.

**Table 4:** Elemental composition in EDX analysis.

Name of the sample	Element	Weight%	Atomic%
**RN**	C K	6.25	34.96
	O K	8.95	37.56
	PbM	84.80	27.48
**SN**	C K	6.45	32.59
	O K	10.78	40.88
	NbL	6.40	4.18
	PbM	76.37	22.36
**JN**	C K	40.90	70.62
	O K	18.95	24.56
	Ca K	1.68	0.87
	As L	0.52	0.14
	Pb M	37.94	3.80
**NB-10**	C K	46.13	70.72
	O K	15.71	18.08
	Na K	−0.01	0.00
	Mg K	1.03	0.78
	S K	7.78	4.47
	Ca K	1.33	0.61
	Fe K	1.36	0.45
	As L	8.45	2.08
	PbM	18.21	2.82
**NB_20**	C K	47.11	67.56
	O K	24.76	26.66
	Na K	1.18	0.88
	Mg K	0.99	0.70
	Ca K	1.31	0.57
	Fe K	0.63	0.19
	As L	9.83	2.26
	Pb M	14.19	1.18
**NB-30**	C K	50.35	75.07
	O K	13.31	14.90
	Na K	0.80	0.62
	Mg K	0.01	0.01
	S K	6.77	3.78
	Ca K	1.93	0.86
	Fe K	0.69	0.22
	As L	14.89	3.56
	Pb M	11.25	0.97
**NB-40**	C K	48.99	67.32
	O K	24.03	24.79
	Na K	0.67	0.48
	S K	6.40	3.29
	Ca K	3.02	1.25
	Fe K	1.38	0.41
	As L	8.68	1.91
	Pb M	6.83	0.54
**NB-50**	C K	71.88	88.60
	O K	7.35	6.80
	Na K	0.09	0.06
	S K	3.50	1.62
	Ca K	1.64	0.61
	Fe K	0.74	0.20
	As L	8.45	1.67
	Pb M	6.35	0.45
**NB-60**	C K	50.79	66.78
	O K	26.87	26.52
	Na K	1.35	0.93
	Mg K	0.91	0.59
	S K	4.16	2.05
	Ca K	0.85	0.33
	Fe K	0.82	0.23
	As L	10.96	2.31
	Pb M	3.29	0.25
**MS-60**	C K	51.02	73.33
	O K	17.02	18.37
	S K	5.43	2.92
	Ca K	0.49	0.21
	Fe K	0.53	0.16
	As L	19.56	4.51
	Pb M	5.96	0.50

### 4.3 Scanning electron microscopy (SEM) interpretation

The whole eight (RN, SN, JN, NB-10, NB-20, NB-30, NB-40, NB-50&NB-60) samples in the SEM images revealed the microlevel was complex with irregular and bulk structure. The trituration of lead with As_2_S_2_ has a slow release rate of S_2_ giving more time for nucleation with lead forming up nanoparticles of lead sulfide crystals. The Steric effect of cysteine which has an acceptor group(COOH) and an electron donor group and a slow release rate of S_2_ are factors that determine the specific internal structure. The low nucleation rate and slow release of S2 also determine the formation of cubic, orthorhombic, hexagonal, monoclinic & and triclinic structures [29].

The triclinic geometry of NB 60 exists in an aggregation of nanoparticles with huge particle sizes. The increment in the particle size also interferes with the reaction with As_2_S_2._ Fig. 3a, Fig. 3b, Appendix A a reveals the presence of very fine nanoparticles to substantiate the aggregation fact. There are narrow pores in the structures ranging from 60 to 100 nm range and the amalgamation of lead with the sour gruel and the As_2_S_2_ can be regarded as the reason. Scanning electron microscopy (SEM) proved the structural pore size deviation (Fig. 3b). Pores of varied sizes were observed, on the surface of NB10, NB20, NB30, NB40, NB50 & NB60 samples were detailed in Table 3 & Fig. 3b. The BET method was used to examine the specific surface area, pore size, and pore volume distribution of NB in a nitrogen adsorption and desorption environment. Table 3 & Fig. 3c lists the sample particulate's surface area, pore size, and volume distribution. The BET surface area for samples JN, NB30, and NB50 was 0.83, 0.726, and 2.98 m2/g respectively. The increase in surface area may increase the chemical rapid reaction be the result of nanoparticle production at high temperatures. Its rapid action is facilitated by its large surface area and varied pore size and pore volume. With rising processing temperature, the pore size distribution was shown by an increase in the area under the hysteresis loops. The surface porosity was varied in NB50 in comparison to *Jarita Naga* (JN). The number and size of pores varies with the increased cycles of incineration. It signifies there is a decrease in mechanical stability and compressive strength. These nanopores have diverse and essential functions that range from maintaining cell homeostasis and participating in cell signaling to activating or killing cells [24]. It can be said that the addition of AS_2_S_2_ to Pb1O1 causes a slow S2– release rate in combination with steric effect, finally changing the compound to Pb11S36 after 60 *puta* ending up in the triclinic structure [25]. Using a mixture of AS_2_S_2_ and sour gruel led to the creation of a product with tiny particles. Sour gruel used as a levigated media in the processing plays a surfactant role and prohibits aggregation of the synthesized particles. Due to the presence of NH2 and H2NNH groups in the sulfur source structure, lone electron pairs can resonate in the sulfur source and stabilize the arsenic structure after S2– release. High S2–release rate led to high nucleation rate and aggregation [30]. Another factor that can be effective on product size and morphology is the steric effect of the sulfur sources. Due to the large structure of AS_2_S_2_, the reaction between the Pb source and AS_2_S_2_ will be limited to a specific direction and triclinic structures are formed. In fact, by decreasing particle size the bandgap is increased. So, the nucleation process is done at various times and different structures and morphologies will be achieved [31]. Also, by decreasing the particle size, minimum energy is required to excite the electron [32].

**Fig. 3a:**
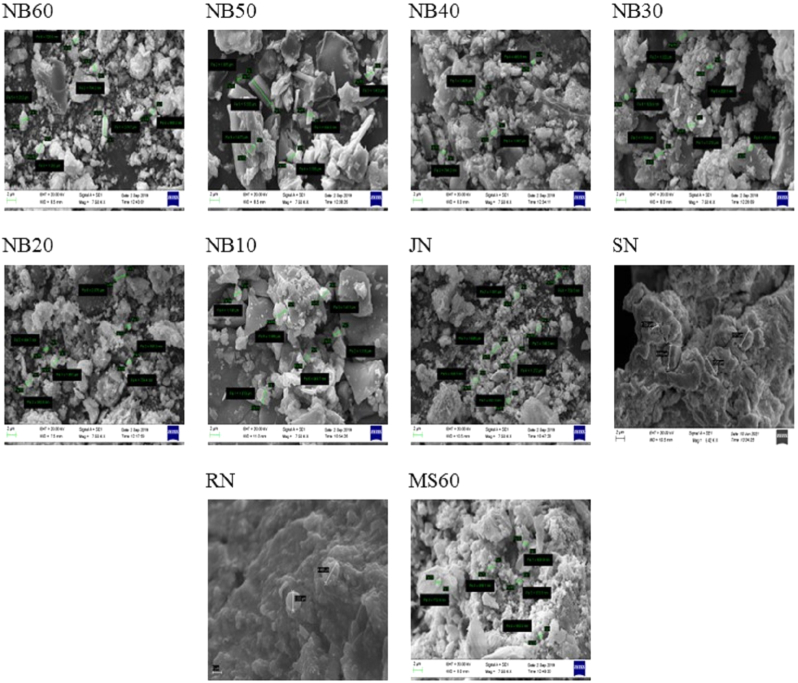
SEM images of NB in different Putas

**Fig. 3b:**
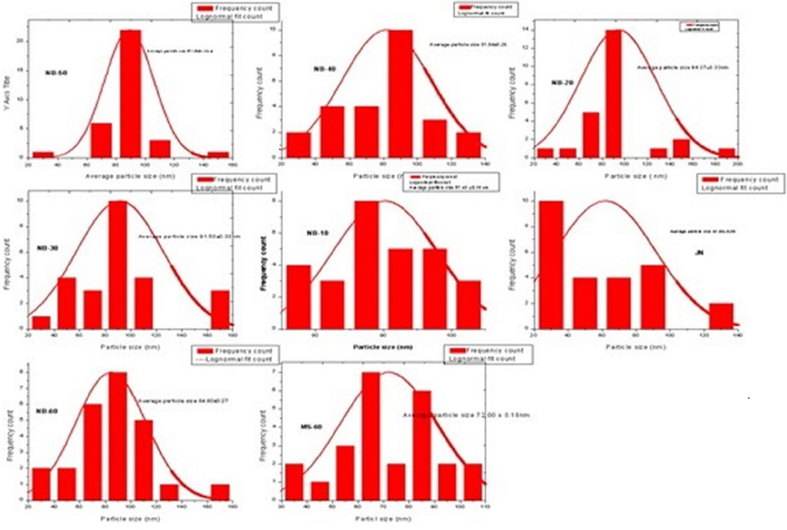
Particle size of NB in different Putas.

The figures of seven samples except RN show the presence of sulfur and the source turns out obvious from the arsenic sulfide addition with each heat treatment and so not evidenced in the raw sample. Thus, the addition of arsenic sulfide, the steric effect, and the slow release of S2 contribute to the final formation of the Pb 11 S36.

### 4.4 Fourier transform infrared (FTIR)

**Fig. 4a:**
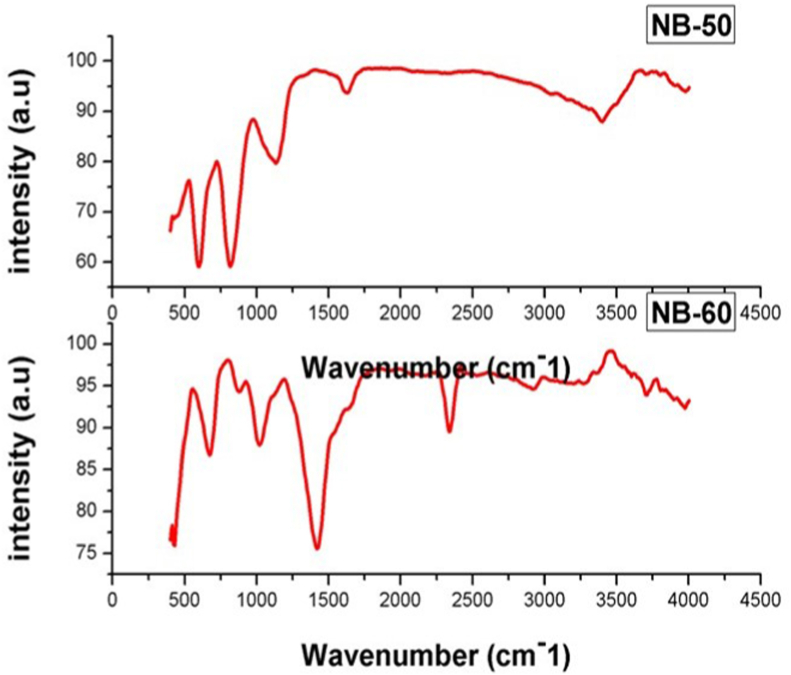
FTIR analysis of NB in different Putas.

Fourier transforms infrared (FTIR) spectra are shown in fig.4a, Table 5. The broad peak around 4000-3500 cm^−1^, 3500-3000 cm^−1,^3300–2500 cm^−1^, 2500-2000 cm^−1^, 1710–1665 cm^−1^, 1400-1000 cm^−1^, 1250–1020 cm^−1^, 1000–650 cm^−1^, 995-985 cm^−1^, 840-790 cm^−1^,730-665 cm^−1^, 600-500 cm^−1^corresponds to C–H, O–H, O–H, CO2, C=O, O–H, C– N, N–H, C–C, C–C, C–C, C–I stretching vibrations. A sharp peak at 3973, 3705 was assigned to vibrations of the Alkanes, Alkenes, and aromatic hydrocarbons. A peak at 3399 cm^−1,^2923.3 cm^−1^ assigned to Carboxylic acids, Alkyl has a role in the pathogenesis of inflammatory disorders within the CNS and possibly other organs. A sharp peak at 1626 is assigned to vibrations of the Alpha, beta–Unsaturated Aldehydes, and Ketones (C=O), while another peak at 1420.8 cm^−1^isrecognized as a stretching vibration of the Carboxylic acid group(O–H). Peaks lying in the 1134.3 are identified as aliphatic amines (C– N) stretching vibrations. A peak around 990 cm^−1^ is identified as Alkene stretching vibrations. Although the FTIR spectra of NB-50 and NB-60 are identical, a well-defined peak around 600 cm^−1^ in NB-60 spectra is assigned to Halo compound(C–I) stretching vibrations [33].

### 4.5 Thermogravimetric analysis (DSC/TGA)

TGA serves as a valuable tool for understanding thermal events associated with *bhasmas* when subjected to heating under predetermined heating rate and temperature conditions. Thermogravimetric analysis curves of NB-10, NB-20, NB-30, NB-40, NB-50, and NB-60 are presented in Table 6 and simultaneously represented in Fig. 4b. The sample was heated from room temperature to 600 °C with a ramp rate of 10 °C min^−1^. Samples of NB as mentioned above starts degrading at 30.78 °C, 20.16 °C, 28.62 °C, 20.33 °C, 35.75 °C, and 22.9 °C respectively. There are four distinct transitions/weight loss associated with the temperature range of room temperature to 600 °C in NB10, NB20, NB30 & and NB40. An initial mass loss of almost 6 %, 1.7 %, 2.3 % & 2.7 % in NB10, NB20, NB30 & NB40 respectively occurs up to 200 °C as shown in Fig. 4b may be due to the removal of water crystallization. This initial mass loss involves multiple transitions occurring during the heating process. The second loss of almost 8.4 %, 12.9 %, 12.2 %, and 16.8 % at 270 °C in NB10, NB20, NB30 & and NB40 respectively has been assigned to the sublimation of sulfur. The third loss of 2.9 %, 7.8 %, 7.2 %, and 5.9 % in the above has been assigned to the sublimation of lead arsenate, which occurs at 440 °C The Final loss of almost 3 %, 4.1 %, 5.1 % in NB20, NB30 & NB40 above 600 °C indicating reaction of lead oxide with tetra arsenic oxide in aqueous dispersion without any mass loss. Thermal decomposition from Pb1 O1 to Pb_5_ O9As3Cl1 occurs in many steps from 22 °C to 600 °C. Decomposition of Pb1 O1 to Pb_1_ O6 occurs above 447.96 °C. Decomposition of Pb_1_ O6 to Pb5 O9As3Cl_1_ occurs above 373.48 °C. The above reaction may be due to the vapourization of water and ethanol content from sour gruel, removal of water of crystallization, and liberation of sulfur and Chlorine at 148.38 °C. Decomposition of Pb1 O4H1As1 to Pb_5_ O9 As3Cl1 above 270 °C forming compounds including arsenic in tetra arsenic oxide and lead compounds. Decomposition of Pb1 O4As1 Cl1 to Pb 11 S36 As 16 at 598.5 °C with endothermic peak producing Pb 11 S36 and As 16 confirmed by small peaks with complete decomposition. Heating of lead oxide with sour gruel (Aqueous dispersion) and tetra arsenic oxide which is formed by the oxidation of arsenic disulfide producing Lead sulfide confirmed by TGA & DTA curve.

In NB-10 no significant mass loss was observed up to 447.96 °C indicating that the sample was free from moisture. The initial thermal decomposition can be seen broadly in a temperature range between 187.70 and 447.96, decomposing Pb_1_O_1_ to Pb_1_O_6_ which may indicate the weak bonds are broken.

In NB-20, in the temperature range of 189.69 °C–373.480C, an exothermic reaction took place indicating symmetrical breaking of crystals that decompose Pb_1_O_6_ to Pb_5_O_9_.

In NB-30, two stages of exothermic reactions were observed. The first occurred in the temperature range from 148.38 °C to 249.57 °C, and the other in the range of 412.7 °C–500 °C decomposes Pb_5_O_9_ to Pb1 O4. It might be the result of intermolecular hydrogen transport.

In NB-40, in between the temperature range 160.82^0^c to 410.80^0^c decomposition of Pb_1_O_4_ to Pb_5_O_9_ takes place in an exothermic fashion indicative of symmetrical break of crystals.

In NB-50, no significant mass loss was observed up to 318.7 °C indicating that the sample was free from moisture. The thermal degradation of NB may be divided into four stages: up to 441.5 °C, about 0.45 % weight loss was noted corresponding to the moisture departure. A notable mass loss (28 %) occurs between 318.7 °C and 441.5 °C due to the decomposition of Pb_1_ O_1_ to Pb_5_O_9_ with a corresponding exothermic peak appearing at 498.2 °C.

For NB-50, DTA/TGA curves were recorded from room temperature (RT) to 598.5 °C in the air with a ramp rate of 10 °C min^−1^. A mass loss of almost 51.30 % occurs up to 598.5C as shown in fig. 4b. This initial mass loss involves multiple transitions occurring during the heating & and trituration process. As observed from the differential thermogravimetric (DTGA) curve of NB-50, dehydration takes place first resulting in the removal of water of crystallization around 119.3C while a hump between 217 and 498.2 °C corresponds to the inorganic compound Pb_5_ O_9_ exothermic nature of transition confirmed by DTA curve. The inorganic compound contains minute quantities of As3Cl1; thermal decomposition from Pb_2_ O_5_ to Pb5O_9_ occurs in many steps from 318.7 °C to 598.5 °C accompanied by endothermic mass losses in designated temperatures [34].

The decomposition of Pb_5_O_9_ to Pb11S36 in NB-60 proceeds as follows. A sharp endothermic peak observed in the DSC curve at 450.9 °C indicates the melting of Pb_1_O_4_ without the involvement of any mass loss as supported by TGA curves. Around 522.7 °C, another endothermic mass loss was observed most probably due to partial evaporation of very small PbO nanoparticles. Fig. 4c shows the DSC/TGA analysis of Pb_11_ S_36_ at a temperature range of 229–522.2 °C with a ramp rate of 10 °C min^−1^. The TGA curve indicates an initial mass loss of about 95.88 % from 148.2 to 322.5 °C. This is confirmed by the DTGA curve which reveals three peaks at 194.6, 322.5, and 468.5 °C, respectively. In the DTA curve, a peak around 148.2 °C corresponds to the exothermic reaction of Pb_11_ S_36_ with oxygen-yielding SO2 as confirmed by a small peak at 404.8 °C in the DTGA curve. TGA graph shows mass gain from 468 to 522.2 °C which is attributed to exothermic reaction at 468 °C producing Pb_11_ S_36_. This is also confirmed by DSC and TGA curves [35].

## 5 Conclusion

*Bhasma* used in the traditional system of *Ayurveda* can be considered as bio nanomedicines, prepared through different steps of specific pharmaceutical techniques affixed to particular metals/minerals [36]. Lead bio nanoparticles as NB is a result of alternate quenching in acid and alkali liquid media (*shodhana*); heating with alkaline herbs (*jarana*); incineration for 60 cycles (*puta*) with arsenic disulfide & sour gruel (*Marana*) forming different crystalline structures of various morphologies through the various stages of preparation. The mentioned process converts the oxide (PbO) form of *naga* into a more complex Pb 11 S36 As 16 form as NB Change of oxide form into sulfide might be a contributable factor in the therapeutic use of this heavy metal together with other factors like the addition of trace elements, functional groups, etc. The particle size reduction can be the total output of pharmaceutical procedures facilitating conversion into lead sulfide. The processing with As_2_ S_2_ makes different crystalline structures that cause the release of S2– with different rates, thus expressing the nucleation process occurring at a differential pace and forming the various morphological structures. The study showed the complexity of the decomposition of changes at different temperatures in different stages of puta. The entire work revealed the complex changes and formation of therapeutically effective combinations as a result of Ayurvedic pharmaceutical processing.

## Sources of funding

This research did not receive any grant from any funding agencies.

## Credit author statement

MKD: Conceptualization, Methodology, Study design, Software, Investigation, Writing – original draft, Writing – original draft, NJ: Visualization, Writing – review and editing, LKD: Conceptualization, Supervision KND: Validation, SD: Formal analysis, RJ: Writing – review and editing

## Declaration of generative AI in scientific writing

No generative AI is used in scientific writing.

## Declaration of competing interest

The other authors declare no conflict of interest.

## References

[1] Mishra G. (2018).

[2] Rajput D., Patgiri B., Galib R., Prajapati P. (2013). Anti-diabetic formulations of Naga bhasma (lead calx): a brief review. Ancient Sci Life.

[3] Maksoodan S., Damodar J., Arya N.C. (1989). Studies on testicular regenertive potential of naga bhasma. Ancient Sci Life.

[4] Islam M.A., Sarkar D.K., Shahinuzzaman M., Wahab Y.A., Khandaker M.U., Tamam N., Sulieman A., Amin N., Akhtaruzzaman M. (2022). Green synthesis of lead sulphide nanoparticles for high-efficiency perovskite solar cell applications. Nanomaterials.

[5] Seshadri S., Saranya K., Kowshik M. (2011). Green synthesis of lead sulfide nanoparticles by the lead resistant marine yeast, Rhodosporidium diobovatum. Biotechnol Prog.

[6] Hou B., Cho Y., Kim B.S., Ahn D., Lee S., Park J.B. (2017). Red green blue emissive lead sulfide quantum dots: heterogeneous synthesis and applications. J Mater Chem C.

[7] Li D., Zhou Z., Sun J., Mei X. (2022). Prospects of NIR fluorescent nanosensors for green detection of SARS-CoV-2. Sensor Actuator B Chem.

[8] Wei S., Guo C., Wang L., Xu J., Dong H. (2021). Bacterial synthesis of PbS nanocrystallites in one-step with L-cysteine serving as both sulfur source and capping ligand. Sci Rep.

[9] Srivastava P., Kowshik M. (2017). Fluorescent lead(IV) sulfide nanoparticles synthesized by Idiomarina sp. strain PR58-8 for bioimaging applications. Appl Environ Microbiol.

[10] Vijaya Bharathi M., Maiti S., Sarkar B., Ghosh K., Paira P. (2018). Water-mediated green synthesis of PbS quantum dot and its glutathione and biotin conjugates for non-invasive live cell imaging. R Soc Open Sci.

[11] Zamani H., Moradshahi A., Jahromi H.D., Sheikhi M.H. (2014). Influence of PbS nanoparticle polymer coating on their aggregation behavior and toxicity to the green algae Dunaliella salina. Aquat Toxicol.

[12] Li D., Li X., Bai J., Liu Y., de Vries R., Li Y. (2021). Rod-shaped polypeptide nanoparticles for siRNA delivery. Int J Biol Macromol.

[13] Nowacek A.S., Balkundi S., McMillan J., Roy U., Martinez-Skinner A., Mosley R.L. (2011). Analyses of nanoformulated antiretroviral drug charge, size, shape and content for uptake, drug release and antiviral activities in human monocyte-derived macrophages. J Contr Release.

[14] Biswas S., Dhumal R., Selkar N., Bhagat S., Chawda M., Thakur K., Gudi R., Vanage G., Bellare J. (2020). Physicochemical characterization of Suvarna Bhasma, its toxicity profiling in rat and behavioural assessment in zebrafish model. J Ethnopharmacol.

[15] Aasy N.K.A., El-Lakany S.A., Masanga P.M., Kamoun E.A., El-Moslamy S.H., Abu-Serie M., Aly R.G., Elgindy N.A. (2023). Concurrent tissue engineering for wound healing in diabetic rats utilizing dual actions of green synthesized CuO NPs prepared from two plants grown in Egypt. Int J Nanomed.

[16] Nanda Harisarana. Bhasma vigyan, first ed. Amritsara: Ayurveda Vigyan Granthamala Karyalaya. p. 313–331.

[17] (2003). Ayurvedic formulary of India, department of Ayush, Ministry of H and FW, Govt of India.

[18] Sharma S. (2012).

[19] Sharma S. (2012).

[20] Sharma S. (2012).

[21] Noyan I.C., Cohen J.B. (1987).

[22] Digges Thomas G., Samuel J., Rosenberg G.W.G. (1966).

[23] Calisir I., Kleppe A.K., Feteira A., Hall D.A. (2019). Quenching-assisted actuation mechanisms in core-shell structured BiFeO3-BaTiO3 piezoceramics. J Mater Chem C.

[24] Kiciński W., Dyjak S. (2020). Transition metal impurities in carbon-based materials: pitfalls, artifacts and deleterious effects. Carbon N Y.

[25] Li C., Lu D., Wu C. (2018). The role of cations in the interactions between anionic N-heterocycles and SO2. Sci Rep.

[26] Elahinia M., Andani M.T., Haberland C. (2014). Shape memory and superelastic alloys. High Temp Mater Mech.

[27] Cheng X. (2014). Tetragonal − orthorhombic − cubic phase transitions in Ag 2 Se nanocrystals. Chem Mat.

[28] Majd S., Yusko E.C., Billeh Y.N., Macrae M.X., Yang J., Mayer M. (2010). Applications of biological pores in nanomedicine, sensing, and nanoelectronics. Curr Opin Biotechnol.

[29] Esmaeili E., Sabet M., Salavati-niasari M., Saberyan K. (2016). Synthesis and characterization of lead sulfide nanostructures with different morphologies via simple hydrothermal. Method.

[30] Devamani R.H.P., Archana M., Maheshwari K., Susmitha D. (2018). Synthesis and characterization of lead II sulphide. Nanoparticles.

[31] Thanh N.T.K., Maclean N., Mahiddine S. (2014). Mechanisms of nucleation and growth of nanoparticles in solution. Chem Rev.

[32] Grant R.S., Coggan S.E., Smythe G.A. (2009). The physiological action of picolinic acid in the human brain.Int. J Tryptophan Res.

[33] Berthomieu C., Hienerwadel R. (2009). Fourier transform infrared (FTIR) spectroscopy. Photosynth Res.

[34] Nafees M., Ikram M., Ali S. (2017). Thermal stability of lead sulfide and lead oxide nano-crystalline materials. Appl Nanosci.

[35] Mcnaughter P.D., Bear J.C., Mayes A.G. (2017). Subject Category : subject Areas : the in situ synthesis of PbS nanocrystals from lead (II) n -octylxanthate within a bisphenol A dimethacrylate sulfur copolymer. R Soc Open Sci.

[36] Joshi N., Dash M.K., Upadhyay C., Jindal V., Panda P.K., Shukla M. (2021). Physico-chemical characterization of kajjali, black sulphide of mercury, with respect to the role of sulfur in its formation and structure. J Ayurveda Integr Med.

