# Supplementary material for: Characterization of lead sulfide obtained from Naga Bhasma
Source: J Ayurveda Integr Med. 2024 Mar 24;15(2):100864. doi: 10.1016/j.jaim.2023.100864 (PMC10979094; doi:10.1016/j.jaim.2023.100864)
Supplement: Multimedia component 1 [file mmc1.docx]

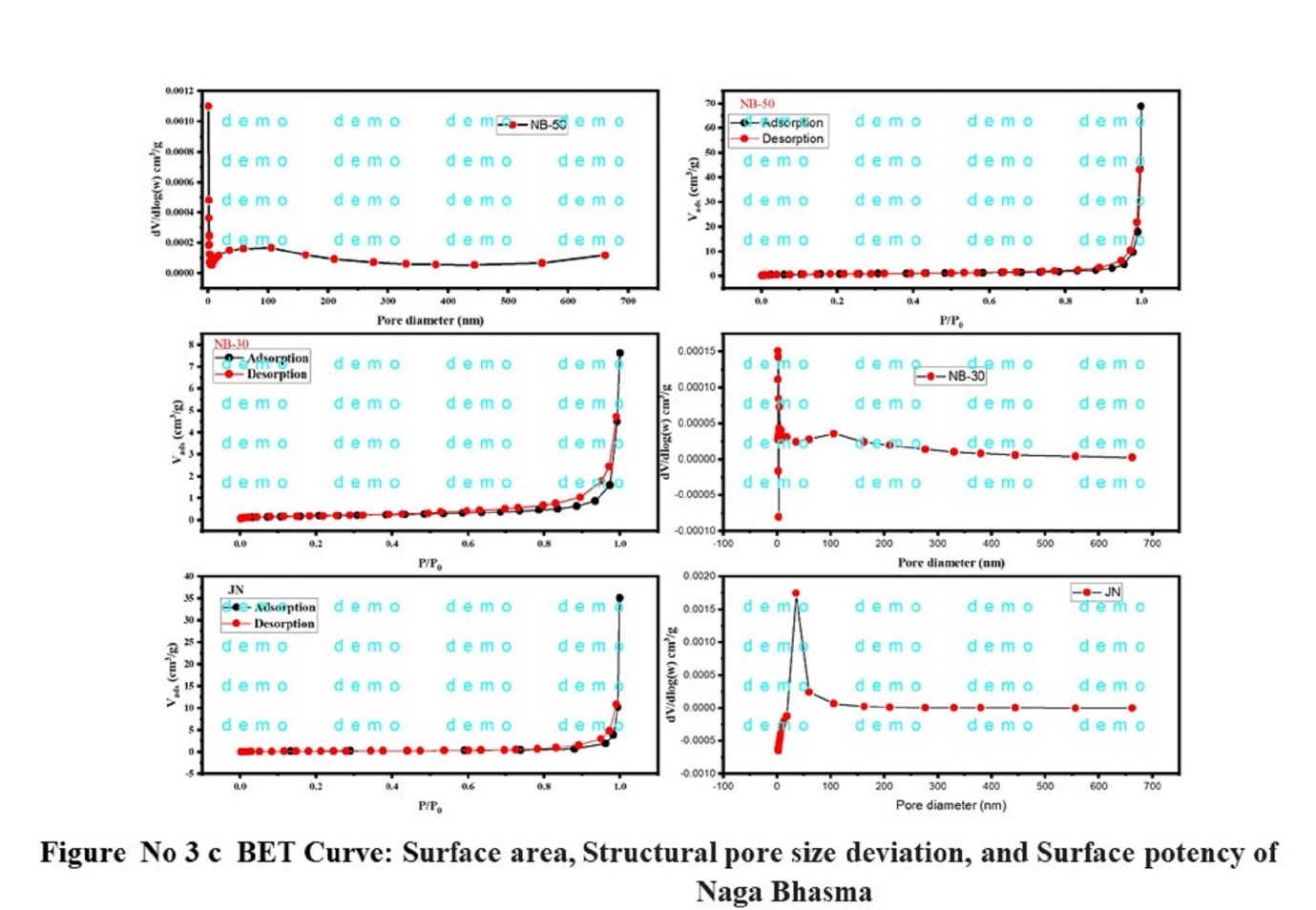


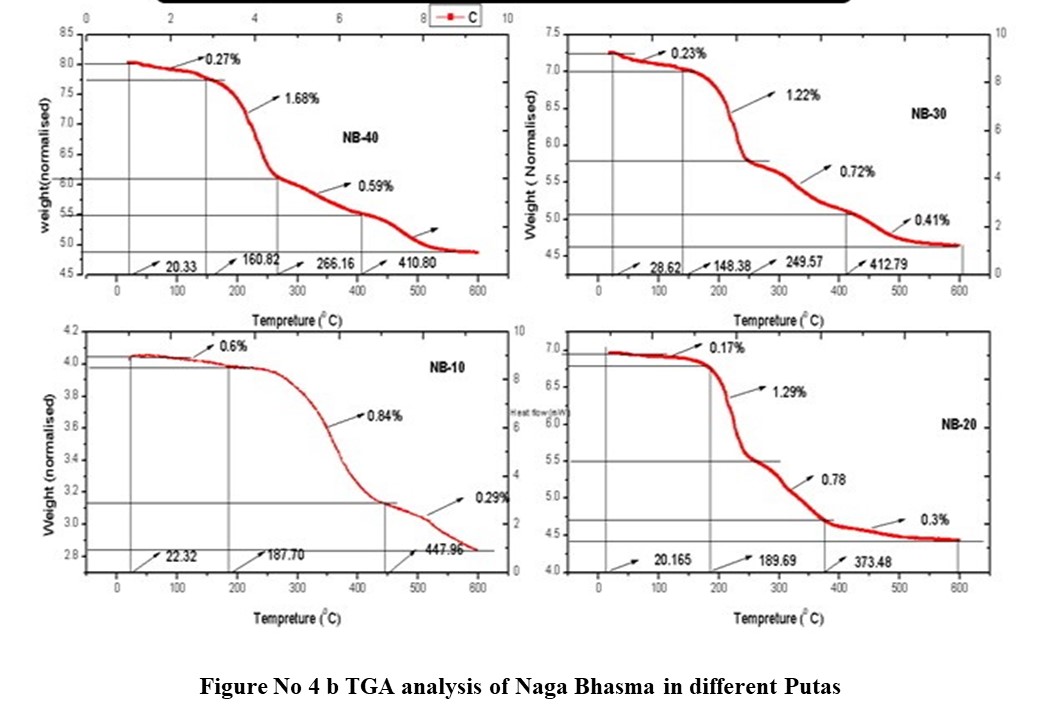


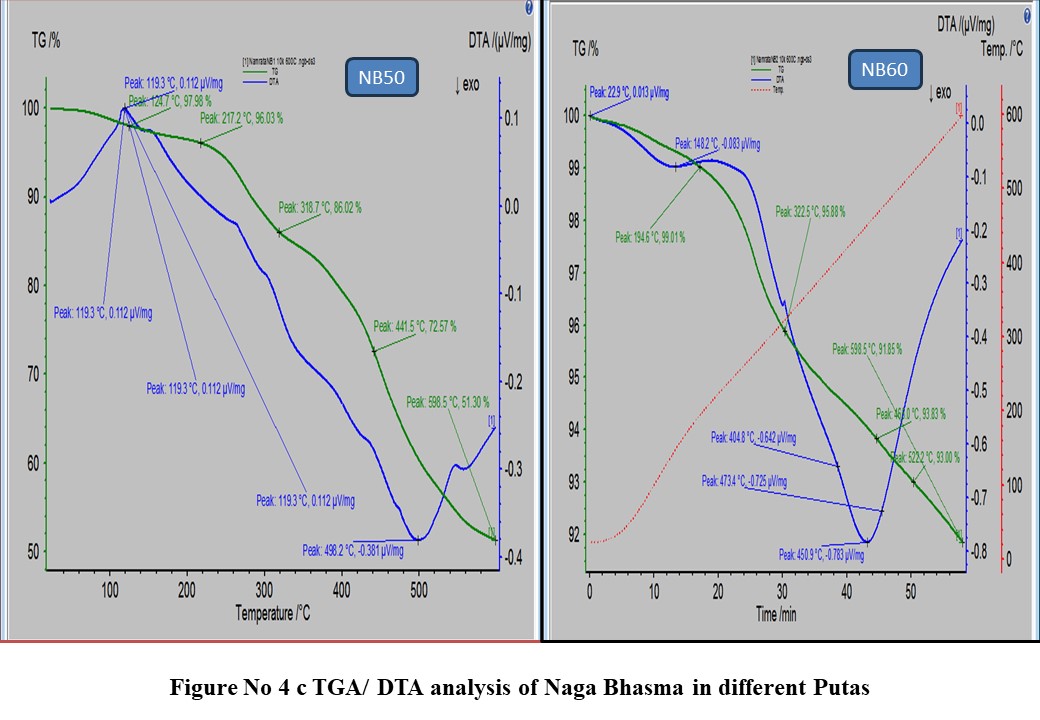


**Table no 2 Interpretation of Powder X-Ray diffraction**

| **Name of the sample** | **Temperature &Total**  **Time** | **hkl/Highest intensity/ Name of the major peaks** | **d (ang) spacing** | **Chemical**  **/Crystal**  **System** | **Calculated density/**  **Average Crystallite Size** | **Shape/Average Particle size** |
| --- | --- | --- | --- | --- | --- | --- |
| RN | 210-230°C  & 4hrs | (004)/26.4  Litharge | 3.20 | PbO/  Cubic | 9.7/  15.8 µm | More Spherical  14.9 µm |
| SN | 350-450°C  & 10hrs | 013/39.5/  Litharge | 3.06 | PbO/  Cubic | 10.4/  35.82 nm | More Spherical/ 35.82 µm |
| JN | 630°-680°C  &4 hrs | (112, 111)/  100  Massicot | 3.06 | Pb1 O1/  Orthorhombic | 9.7/  51.21nm | More Spherical/  51.21nm |
| NB-10 | 700°C at 30 ± 5 minutes  400°C at 30 ± 5 minutes  200°C at 30 ± 5 minutes & 6 hr | (112, 111)/  100  Lead Oxide | 2.77 | Pb_1_ O6/  Orthorhombic | 6.29/  63.77 nm | More Cubic/  63.77nm |
| NB-20 | 700°C at 30 ± 5 minutes  400°C at 30 ± 5 minutes  200°C at 30 ± 5 minutes & 6 hr | (125), (120)/  100  Finnemanite | 2.36 | Pb5 O9  As3Cl1/  Hexagonal | 7.46/  71.65 nm | More Cubic /  66.83nm |
| NB-30 | 700°C at 30 ± 5 minutes  400°C at 30 ± 5 minutes  200°C at 30 ± 5 minutes & 6 hr | (125), (120)  100  Finnemanite | 2.36 | Pb1 O4H1As1/  Hexagonal | 7.46/  67.26 nm | More Oval/  71.65nm |
| NB-40 | 700°C at 30 ± 5 minutes  400°C at 30 ± 5 minutes  200°C at 30 ± 5 minutes & 6 hr | (125), (120)/  100  Finnemanite | 2.36 | Pb_5_ O9  As3Cl1/  Hexagonal | 7.46/  66.83 nm | More Oval/  58.40nm |
| NB-50 | 900°C at 70 ± 5 minutes  700°C at 55 ± 5 minutes  500°C at 80 ± 5 minutes &6 hr | (125), (120)/  100  Schultenite | 2.36 | Pb1 O4  As1 Cl1/  Monoclinic | 7.46/  58.40 nm | More Rod/  67.26nm |
| NB-60 | 900°C at 70 ± 5 minutes  700°C at 55 ± 5 minutes  500°C at 80 ± 5 minutes & 8 hr | (125), (120)  100  Baumhauerite | 1.94 | Pb 11 S36  As 16/  Triclinic | 5.35/  69.06nm | More Rod/  69.06nm |
| MS-60 | 900°C at 70 ± 5 minutes  700°C at 55 ± 5 minutes  500°C at 80 ± 5 minutes & 8 hr | (222)/100  Tetra arsenic Oxide | 3.19 | As4O6/  Cubic | 57.60nm | Rod/  57.60nm |

**Table no 3 BET Curve interpretation of Naga *bhasma***

| Sample Name | BET m^2^/g | Pore size (nm) | Pore Volume (cm^3^/g) |
| --- | --- | --- | --- |
| JN | 0.83 | 116.8 | 0.0537 |
| NB 30 | 0.726 | 48.1 | 0.0097 |
| NB 50 | 2.98 | 69.4 | 0.0631 |

**Table no 5 Functional group in Naga bhasma**

| **S. No** | **Peak** | **Actual Peak** | **Bond** | **Functional group** | **Appearance** |
| --- | --- | --- | --- | --- | --- |
|  | **4000-3500** | **3973** | **C–H** | Alkanes, Alkenes, aromatic hydrocarbons | Short and broad |
|  | **4000-3500** | **3705** | **C–H** | Alkanes, Alkenes, aromatic hydrocarbons | Short and broad |
|  | **3500-3000** | **3399** | O–H | Carboxylic acids, Alkyl | Medium to strong |
|  | 3300–2500 | 2923.3 | O–H | Carboxylic acids, Alkyl | Short and broad |
|  | **2500-2000** | 2339.3 | CO2 | Alkyl | Medium to strong |
|  | 1710–1665 | 1626 | C=O | Alpha,  beta–Unsaturated  Aldehydes, ketones | Strong |
|  | 1400-1000 | 1420.8 | O-H | Carboxylic acid | Strong |
|  | 1250–1020 | 1134.3 | C- N | Aliphatic amines | Often overlapped |
|  | 1000–650 | 878.8 | N–H | Primary Amines, Secondary Amines | Short and broad |
|  | 850–550 | 672.1 | C–Cl or  C–Br | alkyl halides | Medium |
|  | 650-550 | 597.9 | C–Cl or  C–Br | alkyl halides | Medium |
|  | 550-400 | 428.9 | C–Cl or  C–Br | alkyl halides | Medium |

**Table no 6 TGA/DTA Interpretations in *Naga bhasma***

| **Name of the sample** | **Samples start degrading** | **First stage of degradation** | **Second stage of degradation** | **Third stage of degradation** | **Fourth stage of degradation** | **Fifth stage of degradation** |
| --- | --- | --- | --- | --- | --- | --- |
| NB-10 | 30.78 | 187.70 | 447.96 | NA | NA | NA |
| NB-20 | 20.16 | 189.69 | 373.48 | NA | NA | NA |
| NB-30 | 28.62 | 148.38 | 249.57 | 412.79 | NA | NA |
| NB-40 | 20.33 | 160.82 | 266.16 | 410.80 | NA | NA |
| NB-50 | 35.75 | 124.7 | 217.2 | 318.7 | 441.5 | 598.5 |
| NB-60 | 22.9^0^C | 194.6^0^C | 322.5^0^C | 468.0^0^C | 522.2 | 598.5 |
